# Quercetin protects against diabetic encephalopathy via SIRT1/NLRP3 pathway in db/db mice

**DOI:** 10.1111/jcmm.15026

**Published:** 2020-01-30

**Authors:** Tian Hu, Xin‐Yi Lu, Jing‐Jing Shi, Xiao‐Qi Liu, Qu‐Bo Chen, Qi Wang, Yun‐Bo Chen, Shi‐Jie Zhang

**Affiliations:** ^1^ Science and Technology Innovation Center Guangzhou University of Chinese Medicine Guangzhou China; ^2^ Institute of Clinical Pharmacology Guangzhou University of Chinese Medicine Guangzhou China; ^3^ Biological Resource Center The Second Affiliated Hospital of Guangzhou University of Chinese Medicine Guangzhou China; ^4^ Department of Neurology The Second Affiliated Hospital of Guangzhou University of Chinese Medicine Guangzhou China

**Keywords:** diabetic encephalopathy, NLRP3 inflammasome, quercetin, SIRT1

## Abstract

Epidemiological studies have found that diabetes and cognitive dysfunction are closely related. Quercetin has been certified with the effect on improving diabetes mellitus (DM) and cognitive impairment. However, the effect and related mechanism of quercetin on diabetic encephalopathy (DE) are still ambiguous. In this study, we used the db/db mice (diabetic model) to discover whether quercetin could improve DE through the Sirtuin1/NLRP3 (NOD‐, LRR‐ and pyrin domain‐containing 3) pathway. Behavioural results (Morris water maze and new object recognition tests) showed that quercetin (70 mg/kg) improved the learning and memory. Furthermore, quercetin alleviated insulin resistance and the level of fasting blood glucose. Besides, Western blot analysis also showed that quercetin increased the protein expressions of nerve‐ and synapse‐related protein, including postsynapticdensity 93 (PSD93), postsynapticdensity 95 (PSD95), brain‐derived neurotrophic factor (BDNF) and nerve growth factor (NGF) in the brain of db/db mice. Quercetin also increased the protein expression of SIRT1 and decreased the expression of NLRP3 inflammation‐related proteins, including NLRP3, the adaptor protein ASC and cleaved Caspase‐1, the pro‐inflammatory cytokines IL‐1β and IL‐18. In conclusion, the present results indicate that the SIRT1/NLRP3 pathway may be a crucial mechanism for the neuroprotective effect of quercetin against DE.

## 1 INTRODUCTION

Diabetes mellitus (DM) is one of the most prevalent systemic metabolic diseases, which can damage many organs in body.^1^ In worldwide, more than 415 million adults with DM, including 6 million new cases, were counted annually.^2^, ^3^ Many complications caused by diabetes have become the focus of widespread attention. A growing body of evidence suggests that both type 1 diabetes (T1DM) and type 2 diabetes (T2DM) patients exhibit a variety of neuropathological and neurobehavioural changes, including cerebrovascular changes,^4^ insulin signalling systems impairments in cerebral,^5^ poor visual space construction, planning and visual memory injury.^6^, ^7^ Diabetic encephalopathy (DE) is a series of neuropathological changes caused by diabetes, which common symptoms are paraesthesia, numbness and impaired cognition.^8^ The pathogenesis of DE is not completely clear. Persistent inflammation caused by the large secretion of pro‐inflammatory factors is a possible mechanism for DE.^9^ In addition, the insulin signalling pathway is another potential mechanism of DE, which is not only involved in the deposition of amyloid in the brain, but also a neurotrophic factor of nerve cells.^10^ DE has become an important direction in the current research and prevention of diabetes. However, it is still unclear about effective treatment methods and drugs of DE. Therefore, it is urgent to study the pathogenesis of DE and explore new drugs.

Quercetin (3,3′,4′,5,7‐pentahydroxyflavone) is a typical representative flavonoid. Quercetin widely exists in various fruits, vegetables and traditional Chinese medicine plants^6^, ^11^, ^12^; and whose daily intake is about 3‐38 mg/d.^13^ To be a therapeutic agent, quercetin is used to alleviate various diseases, including hepatotoxicity, cardiotoxicity, neurotoxicity and nephrotoxicity.^14^, ^15^ The great effects of quercetin are attributed to its antioxidant^16^ and anti‐inflammatory capacity.^17^ Recently, the combination of dasatinib and quercetin is reported to extend the lives of older people.^18^ Quercetin is an SIRT1 activator.^19^, ^20^, ^21^ Studies have found that quercetin increases monoamine synthesis in aged rats by activating SIRT1 and improves cognitive function in aged rats.^19^ In a diabetic rat model, quercetin can activate SIRT1 and promote glucose and lipid metabolism.^22^ Can quercetin relieve DE through SIRT1? These experimental results provide a reasonable basis for the assumptions of our experiments.^19^, ^22^

SIRT1 (Sirtuin type 1), one of the main members of the sirtuin family, is a deacetylase that targets and regulates the function and activity of the corresponding protein by deacetylation.^21^, ^22^ SIRT1 is thought to play a major role in cell proliferation, differentiation, senescence and apoptosis.^23^, ^24^ Many researches have shown that SIRT1 inhibits the occurrence of inflammatory responses by negatively regulating the NLRP3 inflammasome in vascular endothelial cells.^25^, ^26^ In C57BL/6 mice, quercetin rutin alleviates acute endotoxin‐induced renal injury by inhibiting inflammation and up‐regulating the expression of SIRT1.^27^ In addition, our previous findings suggest that SIRT1 may be the key to improving cognitive function in diabetic mice.^7^ However, whether NLRP3 participates in SIRT1 to improve cognitive function in diabetic encephalopathy mice remains to be further studied.

The nucleotide‐binding domain‐like receptor protein 3 (NLRP3) inflammasome is a multiprotein complex, and it includes the oligomerization of NLRP3, ASC (adaptor protein) and caspase‐1.^28^ And it also plays a very important role in many diseases, including autologous inflammatory disease (CAPS),^29^ multiple sclerosis and lupus,^30^, ^31^ diabetes, acute kidney injury, chronic kidney disease.^32^, ^33^ NLRP3 catalyzes the transformation from inactive pro‐caspase‐1 protein to active caspase‐1protein, and then pro‐IL‐1β and pro‐IL‐18 mature and secrete IL‐1β and IL‐18 under the action of activated caspase‐1 protein.^34^ NLRP3 inflammasome promotes diabetes‐induced endothelial inflammation and atherosclerosis.^35^ Studies have reported that quercetin inhibits NLRP3 inflammatory activation in a rat spinal cord injury model.^36^ Targeting adjustment of the NLRP3 inflammasome protect the nerve damage in spinal cord injury rats.^37^ Previous studies have demonstrated that quercetin can up‐regulate SIRT1 leading to neuroprotection and improve glycolipid metabolism.^19^, ^22^ However, in the DE model, what is the mechanism of action of quercetin? Whether it is related to SIRT1/NLRP3 remains to be verified.

In our study, the db/db mice, a model of T2DM,^7^ were used for study. We explored whether quercetin could improve cognitive dysfunction through NLRP3 signal pathway in db/db mice. We treated our mice with two different doses (35 and 70 mg/kg/d) of quercetin. We finally found the potential mechanism of quercetin of alleviating DE might be through SIRT1/NLRP3 signal pathway.

## 2 MATERIALS AND METHODS

### 2.1 Chemical reagents

**Figure 1:**
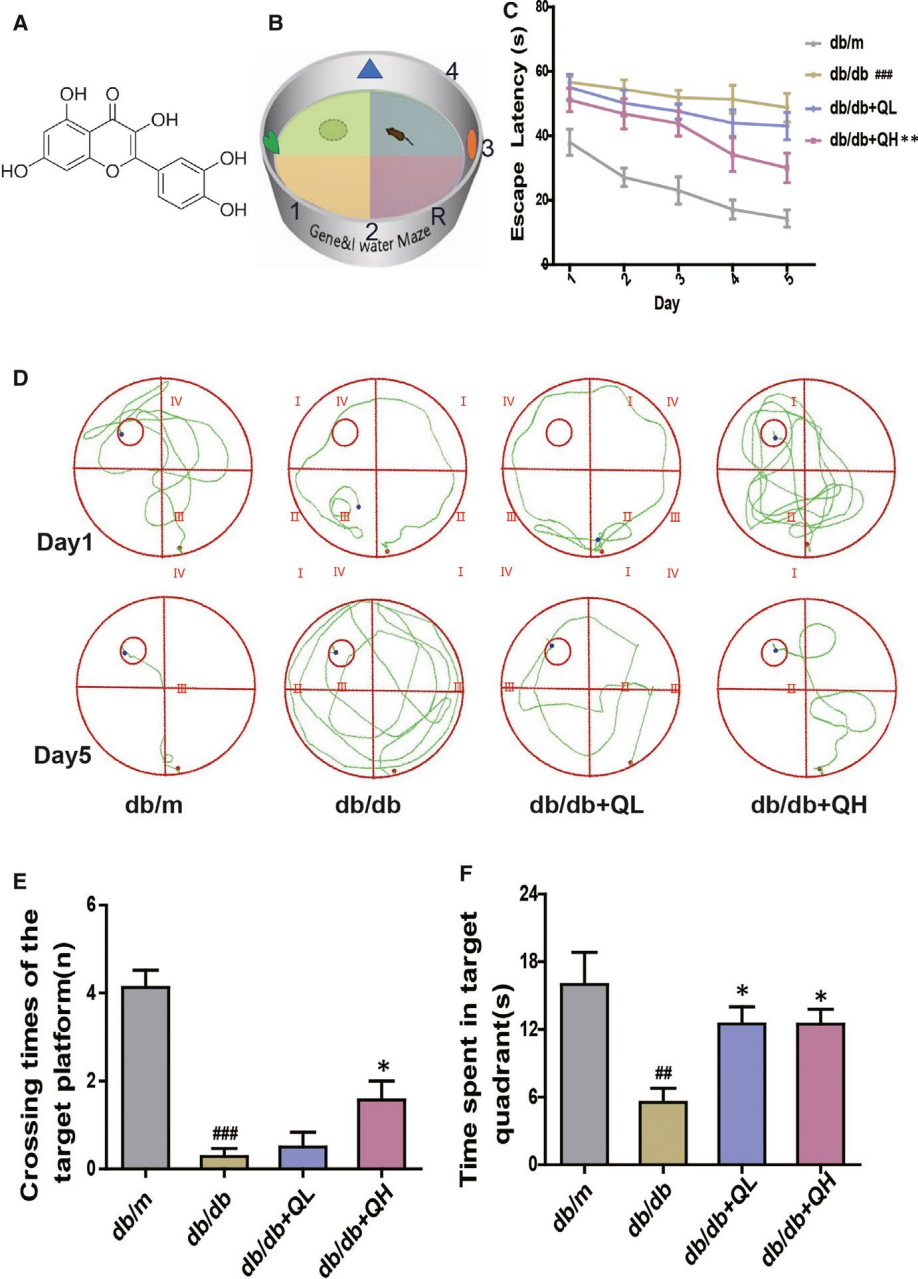
Quercetin ameliorates diabetes‐induced cognitive dysfunction shown by the Morris water maze test in db/db mice. A, The chemical structure of Quercetin. B, Schematic plan of water maze experimental device. C, Escape latency of five consecutive daily tests. D, Swimming paths of the respective groups on the first and fifth day. E, Crossing times of the target platform in the probe trial. F, Time spent in the target quadrant in the probe trial. db/db + QL: Quercetin (35 mg/kg/d); db/db + QH: Quercetin (70 mg/kg/d). Data represent mean ± SEM (n = 8 per group). ^#^ *P* < .05, ^##^ *P* < .01, ^###^ *P* < .001 vs db/m; *P < .05, **P < .01, ***P < .001 vs db/db

Quercetin (98%, Figure 1A) was purchased from Sigma‐Aldrich. Primary antibodies including Postsynapticdensity 93 (PSD93), Postsynapticdensity 95 (PSD95), Nerve growth factor (NGF), SIRT1, ASC, NLRP3 and IL‐1β were purchased from Cell Signaling Technology, Inc. Anti–brain‐derived neurotrophic factor (BDNF), anti‐NLRP3, anti–β‐actin were purchased from Abcam, Inc. Anti–IL‐18 and anti–cysteinyl‐aspartate‐specific proteinase‐1 (Cleaved Caspase‐1) were purchased from Affinity Biosciences. Secondary antibodies (antimouse IgG and anti‐rabbit IgG) were also from CST, Inc.

### 2.2 Animals and treatment

The db/db mice and age‐matched wild‐type C57BL/6J‐db/m mice were purchased from Nanjing Biomedical Research Institute of Nanjing University, Nanjing, China (8 weeks, female). Animals were maintained in SPF animal room, where was provided a 12‐hour light‐12‐hour dark cycle with a relative humidity of 40%‐60% and temperature 22 ± 2°C. The animals were all fed with standard pellet food and freshwater. The animals were randomly arranged into four groups: db/m (0.9% saline, n = 8), db/db (0.9% saline, n = 8), db/db + low dosage of quercetin (QL, 35 mg/kg/d, n = 8) and db/db + high dosage of quercetin (QH, 70 mg/kg/d, n = 8). The treatment cycle is 12 weeks by gavage. The experimental methods applied in our study were conformed to the guide which was promulgated and adopted by the NIH.

### 2.3 Morris water maze test

After 12 weeks of drug treatment, the spatial memory was detected by the Morris water maze test (similar to Morris).^7^, ^38^ Experimental equipment consisted of a black platform, a black circular pool and a record system. The circular pool with a diameter of 120 cm and filled with white and opaque water (30 cm in depth; temperature: 22‐26°C). Furthermore, the pool was divided into 4 symmetrical quadrants. And the escape platform was placed in the central area of appointed quadrant. Animals were conducted an orientation navigation tests for five successive days, and all mice were trained to look for the centre platform before the test.

Furthermore, there were four training trials a day, and the drop location would be changed randomly, recording the time for finding the platform. In this phase, we set up the platform in quadrant IV. The test of time was set as 60 seconds for each trial. When the mouse failed to find the target, it would be there for 10 seconds. Hence, escape latency would be 60 seconds. After the acquisition phase, the centre platform was removed subsequently and mice could swim allodially for 60 seconds to seek for the platform. In this phase, the times of crossing through the centre platform position and all the time spent in the target quadrant suggested the ability of memory retention after learning.

### 2.4 Novel object recognition test

Novel object recognition test was a method for learning and memory test, based on the principle that animals have instinct to explore new objects.^39^ The experimental method was composed of three stages: adaptive phase, orientation phase and finally test phase. The experimental installation was composed of rectangular white box (50 × 25 × 50 cm) and three objects (named A, B and C), of which A was same to B, while the object of C was completely diverse (shape, colour) from the A and B. The test is based on a previous research with minor modification.^40^ On the day 1, the stage of familiarity, mice were acclimated to our test zone (including A and B) for 10 min, then returned to mouse cage. After 24 hours, the mice were placed in the empty box together with the objects of A and B and they would explore for 5 minutes in the test zone. After 24 hours, the B was replaced with C, and animals were also placed back to the box for 5 minutes. In addition, the test zone and objects were washed with 70% ethanol to avoid being affected by the smell after each test. The computer equipment, respectively, recorded the time spent by exploring the novel object (TN) and the familiar object (TF). The time which the mouse spent distinguishing novel and similar objects could be calculated using the identify index (TNI) = (TN − TF)/(TN + TF).^41^, ^42^

### 2.5 Oral glucose tolerance test and insulin tolerance test

Blood glucose levels were measured using the ACCU‐CHEK Advantage glucose analyzer (Roche Diagnostics). The test is based on a previous research with slight modification.^7^ All mice were weighed, and then OGTT was tested after 16 hours fasting. A 2 g/kg glucose solution was given orally by weight; then, glucose levels were tested at 0, 30, 60, 90 and 120 minutes after giving glucose solution by gavage. ITT (insulin tolerance test) was proceeded after three days. The animals were given 0.5 U/kg insulin (Eli Lilly and Co.) in saline by intraperitoneal injection after 4‐hour fasting. Blood glucose levels obtained from the tail vein at appointed time (0, 30, 60, 90 and 120 minutes) Furthermore, AUC, an index of whole glucose excursion after glucose loading, was calculated in according to a previous study.^43^

### 2.6 Nissl's staining

After ITT experiments, all mice were anaesthetized with chloral hydrate (0.04 mL/10 g, Intraperitoneal injection), then they were killed by cervical dislocation. Three paraffin sections of each group were dewaxed in xylene and passed through a series of gradient ethanol and double distilled water rehydrated. Staining was performed according to the Nissl staining kit (Nanjing Jiancheng Bioengineering Research Institute, Nanjing). Images were analysed using an optical microscope and LEICA QWin Plus (Leica Microsystems).

### 2.7 Immunohistochemistry

Three paraffin sections of each group were taken for dewaxing and rehydration. The sections were placed in sodium citrate buffer for antigenic repair for 30 minutes (microwave heating). Blocking with 5% normal goat serum in PBS (37°C, 30 minutes), then incubating with anti‐SIRT1 (1:400; CST) overnight at 4°C. It was washed three times with PBS (10 minutes/time) after rewarming for 30 minutes, and the secondary antibody was added dropwise for 1 hour at 37°C.

### 2.8 Western blot analysis

The tissues of brain were lysed and homogenized in lysis buffer for 15 minutes. The lysed cocktail was centrifuged for 12 minutes (12 000 g, 4°C), and then we measured the protein concentrations using BCA protein assay kit. The total protein (30ug) were separated by SDS‐polyacrylamide gel electrophoresis (SDS‐PAGE) analysis gel and transferred to PVDF membranes. Then, membranes were immersed in 5% skim milk (or BSA) for 1.5 hours at 25°C. The membrane was incubated, respectively, with anti‐NLRP3, anti‐ASC, anti–Caspase‐1, anti–IL‐1β, anti–IL‐18, anti‐PSD93, anti‐PSD95, anti‐BDNF, anti‐NGF and anti‐SIRT1, and mouse anti–β‐actin overnight at 4°C. Then, the membrane was incubated with secondary antibodies for 2 hours. Routinely, a reagent of super‐enhanced chemiluminescence (ECL) made the membrane visualized.

### 2.9 Statistical analysis

Our experimental values were all presented as mean ± SEM. Statistical analyses were all performed using SPSS 19.0 program (IBM). The sample as a whole is normally distributed, and statistical differences in data between groups were performed with one‐way ANOVA, and followed by a post hoc test (Dunnett). *P* < .05 was presented as statistically significant.

## 3 RESULTS

### 3.1 Quercetin relieves cognitive impairment in db/db mice

To investigate whether quercetin could relieve memory and learning impairments, we performed the tests of Morris maze and novel object recognition. In the Morris water maze test, the time for mice to find central platform was decreased gradually in the five testing days (Figure 1B,C). The time for finding the central platform signally prolonged in the db/db group, compared with db/m group. After treated with quercetin (db/db + QL, 35 mg/kg; db/db + QH, 70 mg/kg), animals showed a marked shortness of the escape latency performance, especially for the high‐dose group (Figure 1B).

The swimming path (Figure 1C): Animals swimmed irregularly in designated areas on the first day. After five days of training, the trajectory of db/db showed a long and disorderly swimming path, which was improved by quercetin treatment, especially in high‐dose group (Figure 1C). After the removement of the platform on the sixth day, the group of db/db had shorter crossing times and target quadrant dwelling time than the group of db/m (Figure 1D,E). The groups of quercetin had longer platform crossing times and target quadrant dwell time than those in the db/db group, notably for db/db + QH group (Figure 1D,E).

**Figure 2:**
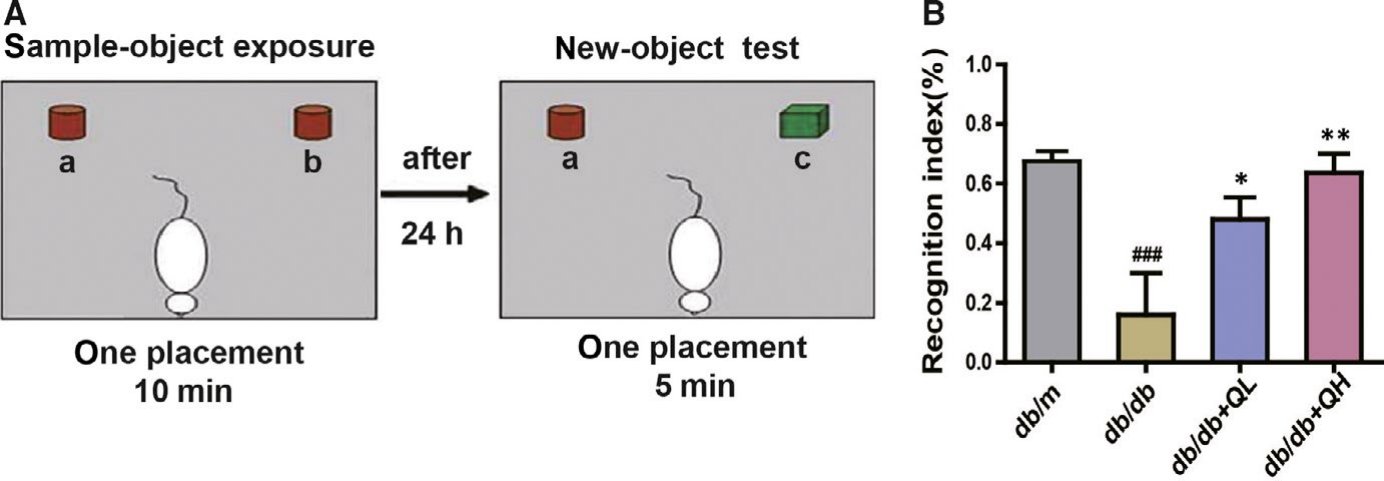
Quercetin prevents learning and memory impairments by the novel object discrimination in db/db mice. A, Schematic diagram of new object recognition experimental device. B, Recognition index (TNI) = (TN − TF)/(TN + TF). db/db + QL: Quercetin (35 mg/kg/d); db/db + QH: Quercetin (70 mg/kg/d). Data represent mean ± SEM (n = 8 per group). ^#^ *P* < .05, ^##^ *P* < .01, ^###^ *P* < .001 vs db/m; *P < .05, **P < .01, ***P < .001 vs db/db

In the novel object recognition test (Figure 2A), the db/db group showed signally lower level of the TNI than db/m group. After treated with quercetin, db/db + QH group show significantly a higher level than db/db group (Figure 2B).

### 3.2 Quercetin reduces fasting glucose and insulin resistance in db/db mice

**Figure 3:**
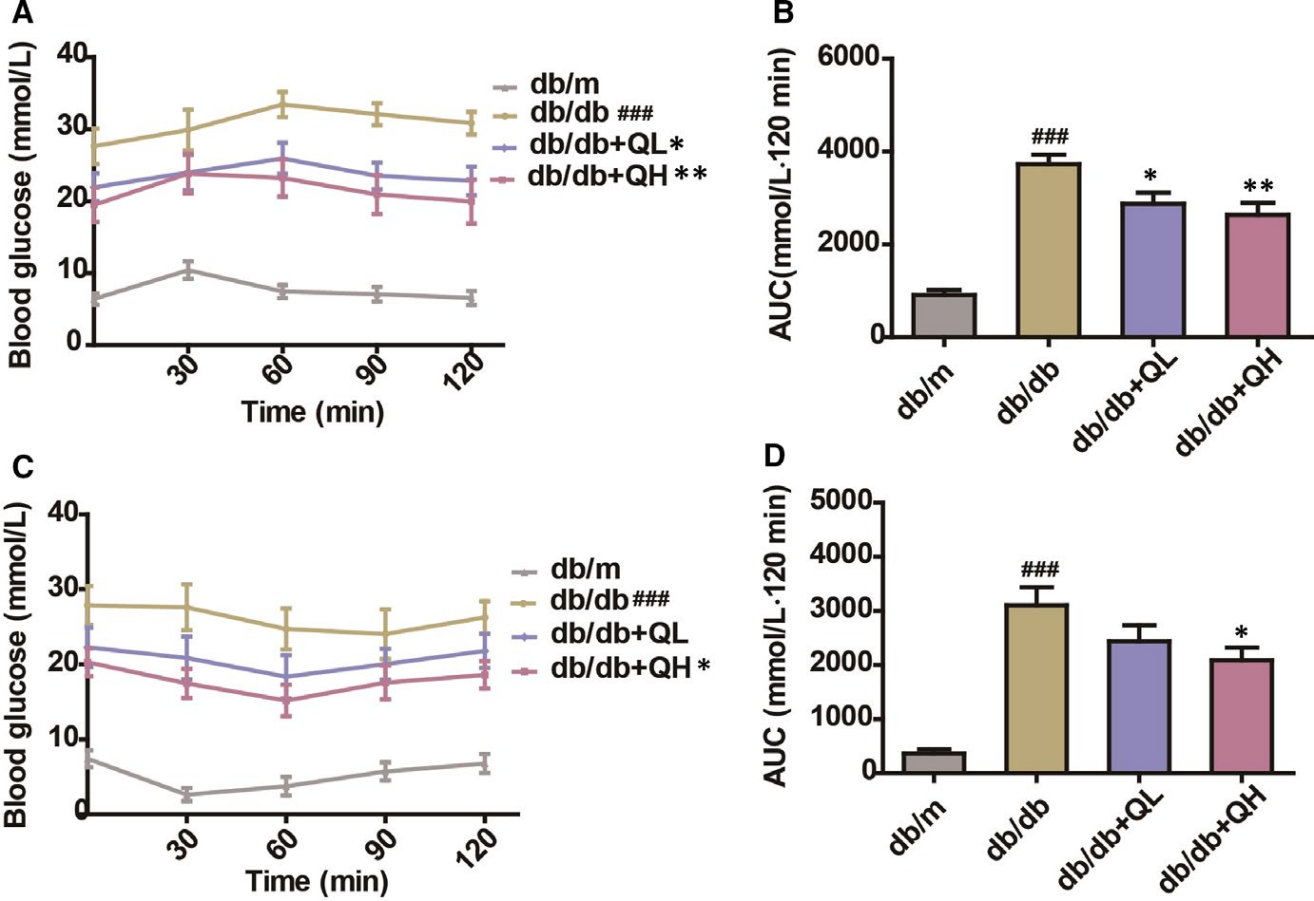
Quercetin decreases fasting glucose in db/db mice. A, OGTT. B, AUC (OGTT). C, ITT. D, AUC (ITT). db/db + QL: Quercetin (35 mg/kg/d); db/db + QH: Quercetin (70 mg/kg/d). Data represent mean ± SEM (n = 8 per group). ^#^ *P* < .05, ^##^ *P* < .01, ^###^ *P* < .001 vs db/m; *P < .05, **P < .01, ***P < .001 vs db/db

In OGTT test, the db/db group showed a higher peak of glucose rise and a slower reduction of blood glucose concentration than db/m group. The db/db group manifested glucose intolerance because of the obviously high glucose excursion, and it is AUC during the OGTT (Figure 3A,B). After treated with quercetin (db/db + QL, 35 mg/kg; db/db + QH, 70 mg/kg), the rising peak of mice glucose became lower and the decreasing concentration of blood glucose was faster than db/db group, especially for high‐dose group (Figure 3A).

In ITT test, the group of db/db mice exhibited insulin resistance, compared with db/m group. The db/db group showed a slower rate of blood glucose concentration decline than db/m (Figure 3C). After treated with quercetin, the blood glucose concentration was significantly lower and insulin resistance was significantly improved compared with db/db group, notably for db/db + QH group (Figure 3C,D).

### 3.3 Quercetin improves neurodegeneration in db/db mice

**Figure 4:**
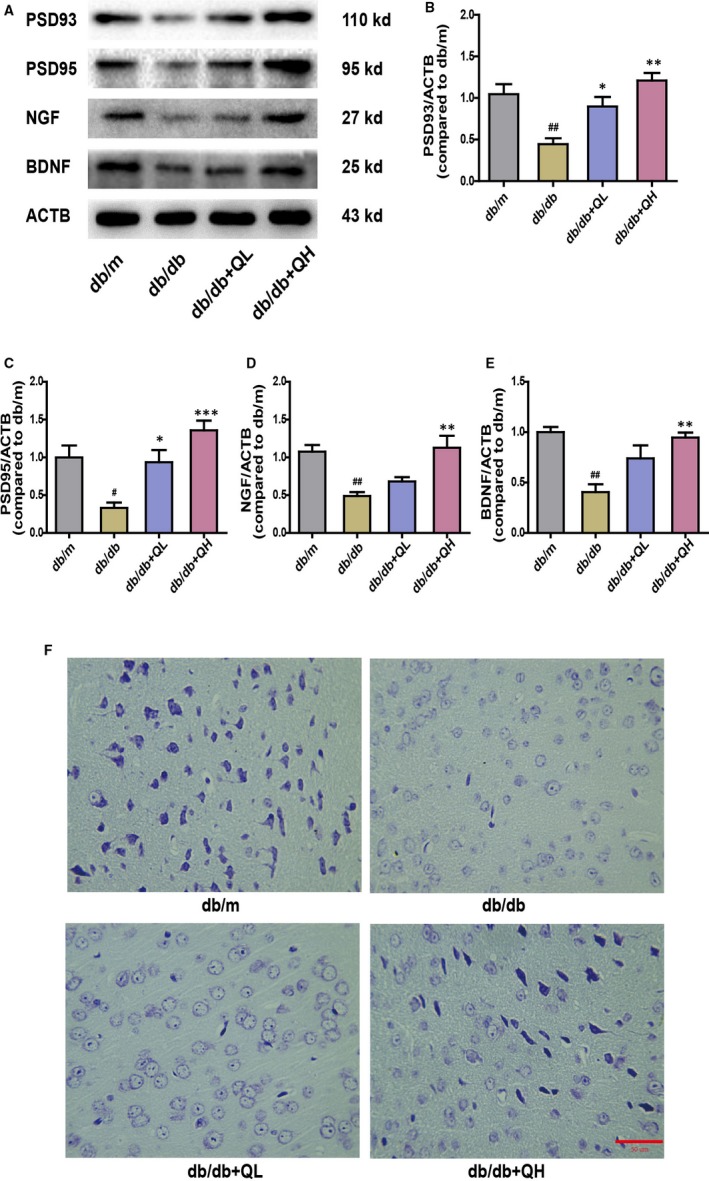
Quercetin increases neurotrophic factor levels in the brain of db/db mice. Representative Western blot results A, of protein expression in the brain of db/db mice. B, PSD93. C, PSD95. D, NGF. E, BDNF. F, Nissl's staining in cortex. db/db + QL: Quercetin (35 mg/kg/d); db/db + QH: Quercetin (70 mg/kg/d). Data represent mean ± SEM (n = 8 per group). ^#^ *P* < .05, ^##^ *P* < .01, ^###^ *P* < .001 vs db/m; *P < .05, **P < .01, ***P < .001 vs db/db. Bar: 50 μm

As illustrated in Figure 4, the protein expressions of the neurotrophic factors, including PSD93, PSD95, NGF and BDNF, were sharply decreased in db/db group (Figure 4A‐E). After treatment with quercetin, especially for high‐dose group, the levels of PSD93, PSD95, BDNF and NGF were increased in the brain (Figure 4A‐E). In addition, the results of Nissl staining further confirmed the above changes (Figure 4F). The number of Nissl bodies in the db/db mice was significantly reduced compared with the db/m mice, and the staining was observably shallow. After treated with quercetin, the number and colour of Nissl bodies were significantly improved. These results indicate that quercetin could improve neurodegeneration in db/db mice.

### 3.4 Quercetin activates SIRT1 and inhibits NLRP3 inflammasome activation

**Figure 5:**
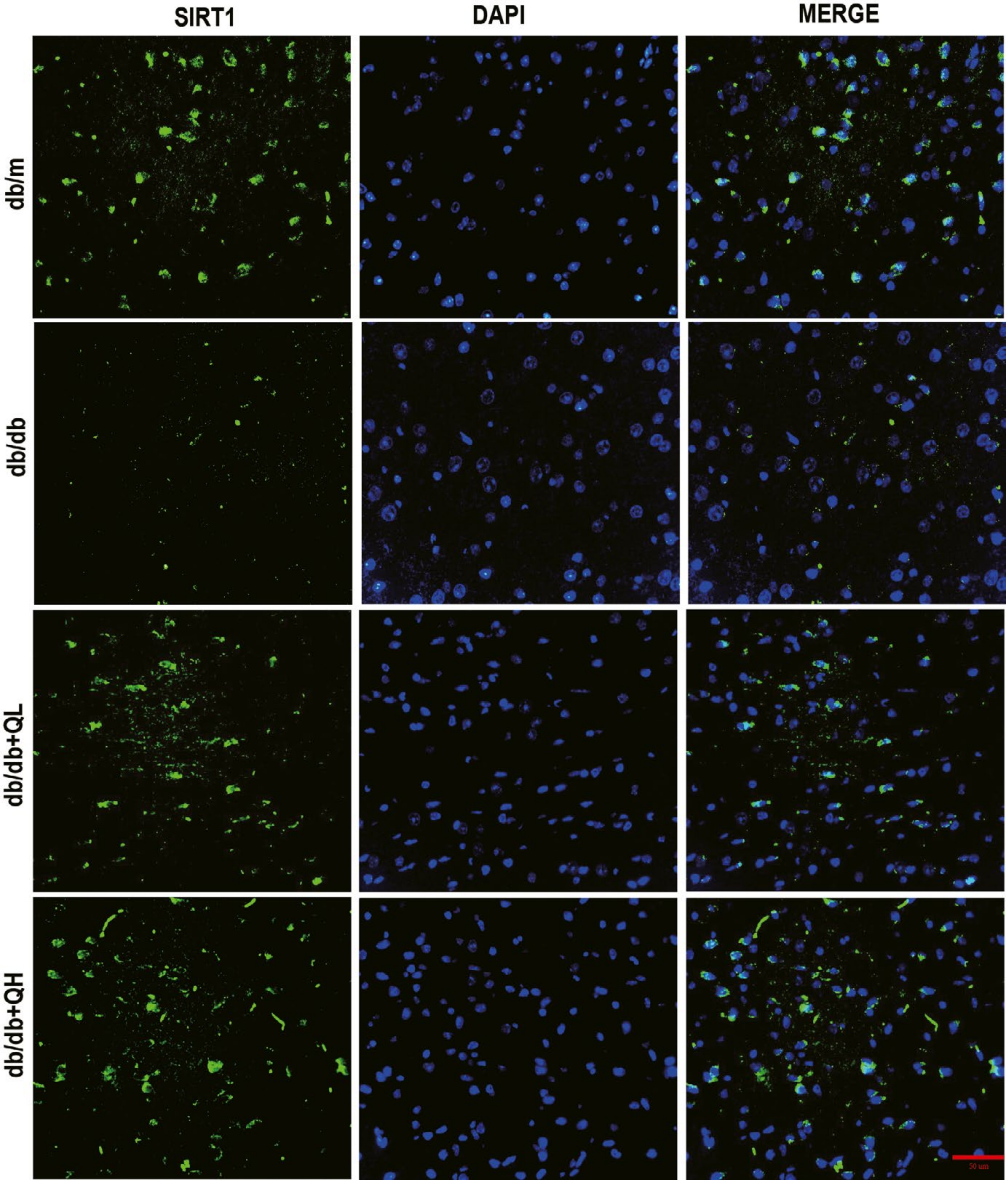
Quercetin activates SIRT1 in the brain of db/db mice. Immunofluorescence of SIRT1 in cortex. Bar: 50 μm

**Figure 6:**
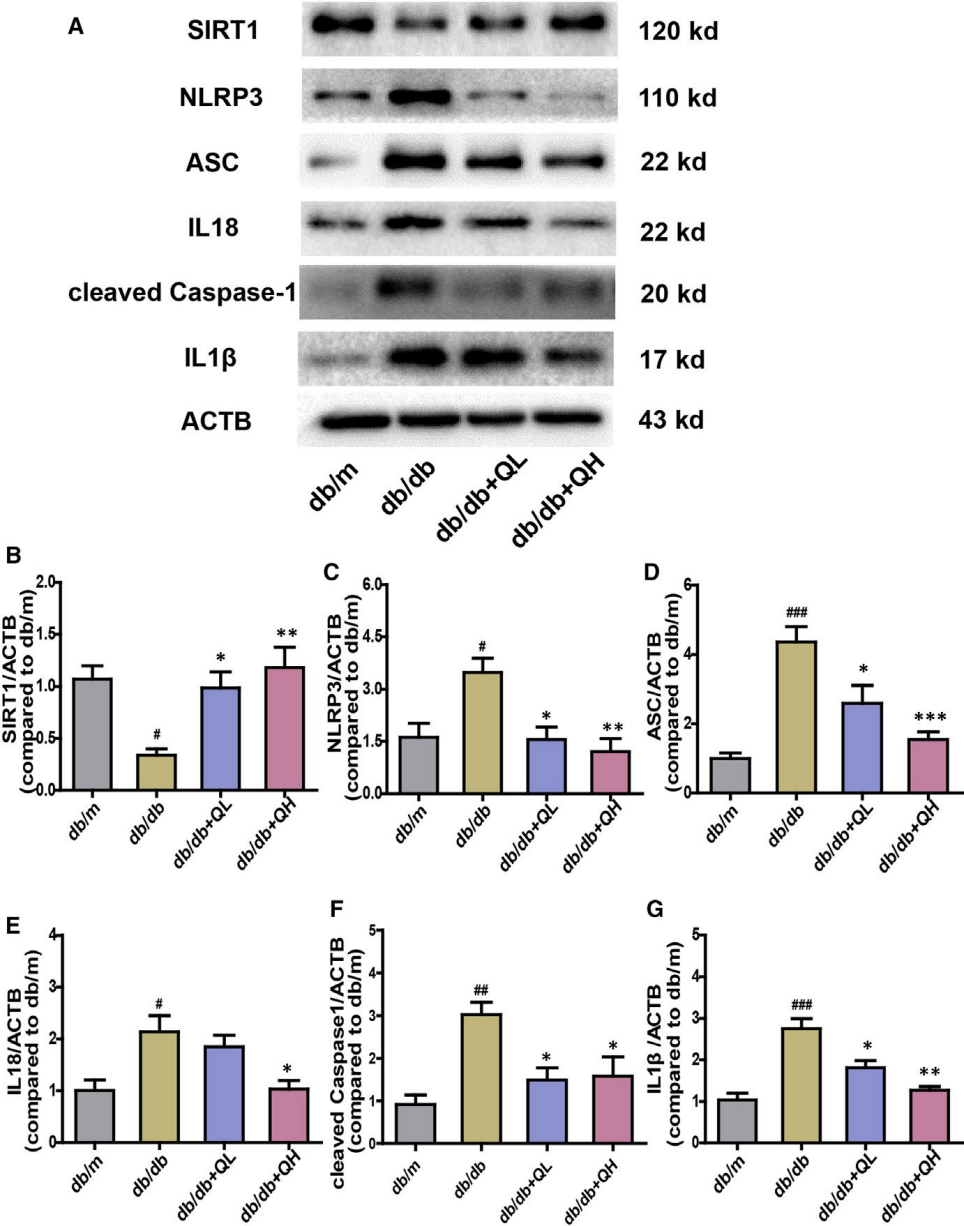
Quercetin activates SIRT1 and inhibits NLRP3 inflammasome activation in the brain of db/db mice. Representative Western blots A, results of protein expression in the brain of db/db mice. B, SIRT1. C, NLRP3. D, ASC. E, IL‐18. F, Cleaved Caspase‐1. G, IL‐1β. db/db + QL: Quercetin (35 mg/kg/d); db/db + QH: Quercetin (70 mg/kg/d). Data represent mean ± SEM (n = 8 per group). ^#^ *P* < .05, ^##^ *P* < .01, ^###^ *P* < .001 vs db/m; *P < .05, **P < .01, ***P < .001 vs db/db

As illustrated in Figures 5 and 6, the db/db group showed a lower protein expression of SIRT1 than db/m (Figures 5 and 6A,B). After treatment with quercetin, especially for high‐dose group, the protein expression of SIRT1 was increased (Figures 5A and 6B). Subsequently, we measured the expression levels of NLRP3 inflammation‐related proteins, including NLRP3, cleaved Caspase‐1(p20), ASC, IL‐1β and IL‐18, which were evident different between groups of db/m and db/db (Figure 6C‐G). After treatment with quercetin, the expression of these proteins expression was sharply decreased, contradistinguished with db/db group (Figure 6C‐G). The data indicated quercetin could activate SIRT1 and inhibit NLRP3 inflammasome activation to protect DE.

## 4 DISCUSSION

In our study, we proved that quercetin could relieve diabetes‐associated cognitive impairment in db/db mice. The experimental results found that quercetin preserved learning and memory, alleviated insulin resistance and decreased blood glucose in db/db mice. For mechanism exploring, quercetin increased nerve and synapse‐related protein expression and reduced the protein expression of neuroinflammatory factors in the brain of db/db mice. Furthermore, quercetin activated SIRT1 and inhibited the expressions of NLRP3‐regulated inflammation‐related proteins, which might be the key mechanisms of the neuroprotective effect of quercetin.

Diabetes is a key cause of cardiovascular disease, retinal disease, and the development and progression of neurological diseases.^44^ DE is a series of behavioural and pathological changes caused by constitutive hyperglycaemia, including cognitive decline, neuronal loss and disorders of glycolipid metabolism.^45^, ^46^, ^47^ DE decreased the patient's ability of the learning and memory.^48^ In previous studies, quercetin has been shown to lower blood lipid levels and increase glucose tolerance.^49^ Recently, studies have reported that quercetin has neuroprotective effects on cognitive impairment caused by diabetes.^50^ In our study, using db/db mice showed the neuroprotective mechanisms of quercetin. Behavioural results (Morris water maze test, new object recognition test) showed that quercetin (70 mg/kg) significantly improved learning and memory levels. At the same time, quercetin reduces insulin resistance and promotes glucose metabolism by reducing the susceptibility to T2D/IR. The therapeutic effect of quercetin on DE is clearly consistent with previous studies.^49^, ^51^, ^52^

The activation of NLRP3 inflammatory bodies is closely related to the pathogenesis of DM.^53^, ^54^ In a previous study, NLRP3 inflammasome is activated in the neurons of hippocampus in db/db mice.^55^ NLRP3 catalyzes the activation of caspase‐1, thereby promoting the maturation and secretion of IL‐1β and IL‐18.^7^, ^56^ Studies have found that inhibiting NLRP3 inflammasome activation can reduce IL‐1β levels in hippocampus of DM rats,^57^ and the expression level of hippocampal IL‐1β is related to cognitive function in DM mice.^55^ Quercetin has been shown to increase microglial activation and potently inhibit pro‐inflammatory factors.^58^ In addition, quercetin attenuates NLRP3 inflammatory activation and lipid accumulation in diabetic rats.^59^, ^60^, ^61^ In our study, we found that the NLRP3 pathway is activated in the brain of db/db mice and that synapses and trophic factors are significantly reduced. This effect of quercetin on neuroinflammation is based on previous studies.^62^, ^63^, ^64^, ^65^

SIRT1 regulates intracellular signalling molecules, inhibits apoptosis, regulates inflammation and resists oxidative stress.^66^, ^67^ SIRT1 can be involved in regulating the formation of Alzheimer's disease amyloid and maintaining the stability of the neuronal genome.^68^, ^69^ Studies have reported that quercetin improve lipid, glucose metabolism and inhibits neurodegeneration through SIRT1 signalling pathway.^22^, ^70^ These results suggest SIRT1 might play a pivotal role of quercetin on DE in db/db mice. In addition, SIRT1 is closely related to the activation of NLRP3 inflammasome.^71^ In rat cerebral ischaemia/reperfusion models and ventilation‐induced lung injury models, SIRT1‐dependent inhibition of NLRP3 inflammatory body activation.^72^, ^73^ Therefore, it may be assumed that quercetin effects on DM in db/db mice through SIRT1/NLRP3 signalling pathway. In our study, we found that SIRT1 were observably decreased when the NLRP3 increased in the brain of db/db mice. This effect of quercetin on SIRT1/NLRP3 signalling pathway is according with previous studies.^19^, ^22^, ^74^

## 5 CONCLUSION

Quercetin may ameliorate DE by decreasing fasting glucose, up‐regulating activity and protein level of SIRT1, and inhibiting the expressions of NLRP3‐regulated inflammation‐related proteins. Quercetin shows the potential for the prevention and therapy of DE. However, further evidence is still needed to confirm this phenomenon. These data could be useful for explaining the underlying neuroprotective mechanisms of quercetin on DE.

## CONFLICTS OF INTEREST

The authors declare that there is no conflict of interest.

## AUTHOR CONTRIBUTIONS

S.‐JZ designed the study. HT, J.‐JS and X.‐QL conducted the experiment. S.‐JZ and HT contributed to initial data analysis and interpretation and drafted the initial manuscript. X.‐Y.L and Q.‐B.C helped revised the manuscript. QW, S.‐JZ and Y.‐BC supervised all aspects of the study, critically reviewed and revised the manuscript, and approved the final manuscript as submitted.

## Data Availability

The data used to support the findings of this study are available from the corresponding author upon request.
